# Metabolic alkalosis: a new red flag in status epilepticus

**DOI:** 10.1007/s00415-024-12603-x

**Published:** 2024-07-27

**Authors:** Francesco Misirocchi, Hervé Quintard, Margitta Seeck, Pia De Stefano

**Affiliations:** 1 Unit of Neurology, Department of Medicine and Surgery, University of Parma, Parma, Italy; 2 Neuro-Intensive Care Unit, Department of Intensive Care, University Hospital of Geneva, Geneva, Switzerland; 3 Medical Faculty of the University of Geneva, Geneva, Switzerland; 4 EEG & Epilepsy Unit, Department of Clinical Neurosciences, University Hospital of Geneva, Geneva, Switzerland

**Keywords:** Status epilepticus, Outcome, Intensive care, pH, Alkalosis

## Abstract

**Background:**

Status epilepticus (SE) is a heterogeneous neurological emergency with significant variability in prognosis, influenced by underlying disease and pathophysiological context. Acid–base disturbances are common in critically ill patients, yet their distribution and impact in SE patients remain poorly understood.

**Methods:**

This was an observational cohort study including non-hypoxic SE patients with available blood gas analysis within the first 24 h of SE, treated at the University Hospital of Geneva, Switzerland between 2015 and 2023. Acid–base disturbances were classified using the Henderson–Hasselbalch equation, with prevalent metabolic alkalosis confirmed through the Stewart approach. Primary outcomes were in-hospital mortality, Glasgow Outcome Scale (GOS) at discharge, and return to premorbid neurologic function.

**Findings:**

Among 540 SE patients, 365 were included. Half of patients exhibited acid–base disturbances within the initial 24 h of SE, with metabolic and respiratory acidosis being the most prevalent, though not prognostically significant. After correction for possible confounders, metabolic alkalosis (6%) was associated with increased in-hospital mortality (*P* = 0.011; OR = 4.87, 95% CI = 1.29–7.84), worse GOS (*P* = 0.012; OR = 3.18, 95% CI = 1.29–7.84), and reduced likelihood of returning to premorbid function (*P* = 0.017; OR = 3.30, CI95% = 1.24–8.80). Following the Stewart approach, 9% of patients had predominant metabolic alkalosis, associated with worse GOS (*P* = 0.005; OR:3.37, 95%CI = 1.45–7.82), and reduced chance of returning to baseline (*P* = 0.012; OR = 3.29, CI95% = 1.30–8.32). Metabolic alkalosis was related to hypoalbuminemia and lower serum potassium.

**Conclusion:**

Metabolic alkalosis strongly predicts mortality and adverse functional outcome in SE patients. Prospective studies should assess whether early detection and correction of metabolic alkalosis and related electrolyte imbalances can improve SE prognosis.

## Introduction

Status epilepticus (SE) is a neurological emergency with ongoing epileptic seizures [1]. This condition is highly heterogeneous, and it is often described as a complication of other acute or chronic insults rather than a disease itself [2].

Prognosis in SE is highly variable, ranging from severe neurological disability to death [1, 3, 4]. The outcome of SE is determined by the underlying disease but is also heavily influenced by the pathophysiological context in which it develops, including both factors independent from SE (such as age, comorbidities, concomitant therapy) and/or triggered by SE (such as organ, metabolic, and electrolytic dysfunctions) [5]. This bidirectional relationship makes prognostication particularly challenging [2, 6].

It has been demonstrated that low brain and blood pH levels strongly suppress epileptic activity [7, 8], while emerging evidence suggests that alkalosis may trigger febrile and hypoxic-related seizures [9, 10]. However, few studies have systematically examined acid–base disorders and their association with outcome in SE patients [11].

Notably, pH alterations are common in critically ill patients and can substantially impact patients’ outcome [12]. SE may cause multiple acid–base disturbances, including respiratory acidosis due to impaired respiratory function and metabolic acidosis from anaerobic muscle metabolism [11, 13, 14].

Our study aims to investigate acid–base disturbances in a large and heterogeneous SE population, examining the relationship between acid–base disorders, functional outcomes, and mortality.

## Methods

### Data collection and definitions

This is an observational cohort study performed at the University Hospital of Geneva (HUG), a Swiss academic tertiary medical care center. The STROBE guidelines were followed to improve the quality of the study [15].

Data from all adult patients (aged ≥ 18 years) treated for SE between November 1st, 2015 and December 31st, 2023 were retrospectively identified from a SE registry retrospectively collected to October 2021, and prospectively collected from November 2021 to 2023.

Data were collected and managed with the password encrypted online browser-based, metadata-driven database organizer REDCAP (Research Electronic Data Capture) [16].

Patients with SE following cardiorespiratory arrest and patients without blood gas analysis (BGA) during the first 24 h of SE were excluded.

The following features were retrieved: patients’ age, sex, comorbidities, SE etiology, semiology, and duration. SE types were defined as recommended by the International League Against Epilepsy (ILAE) [1]. SE etiology was defined as acute symptomatic, remote symptomatic, progressive symptomatic, and unknown [1]. SE etiology was categorized as potential non-fatal and fatal, following previous reports [17].

Charlson Comorbidity Index (CCI, range 0–37) and the Status Epilepticus Severity Score (STESS, range 0–6) were calculated to quantify comorbidity burden and illness severity, respectively [18–20].

Sepsis has been defined in the presence of established infection together with organ dysfunction, represented by an increase in the Sequential (sepsis-related) Organ Failure Assessment (SOFA) score of 2 points or more [21, 22].

SE duration was defined as the period between SE diagnosis and the clinical and/or EEG evidence of seizure termination, as described elsewhere [23].

The Glasgow Outcome Scale (GOS, range 1–5) was used to assess outcome at discharge, dichotomized as follows: bad outcome—GOS of 1 to 3; good outcome—GOS of 4 to 5 [24].

Values of pH, partial pressure of carbon dioxide (PaCO_2_) and bicarbonate (HCO_3–_) from BGA during the first 24 h of SE were gathered. The following laboratory data within the first 24 h of SE were also collected: serum albumin [g/L], serum creatinine [umol/L], serum phosphate [umol/L], serum bilirubin [umol/L], serum urea [umol/L], serum sodium [mmol/L], and serum potassium [mmol/L].

Values obtained beyond 24 h from SE onset were excluded. For patients with multiple laboratory values within the first 24 h, only the first assessment was considered.

According to the Henderson–Hasselbalch equation [25], the following acid–base disturbance categories were defined: acidosis (either Respiratory [pH < 7.35, PaCo_2_ > 45 mmHg], metabolic [pH < 7.35, HCO_3_ < 22 mmol/L] or mixt [pH < 7.35, PaCo_2_ > 45 mmHg, HCO_3_ < 22 mmol/L]), normal pH (pH 7.35–7.45) and alkalosis (either respiratory [pH > 7.45, PaCo_2_ < 35 mmHg], metabolic [pH > 7.45, HCO3 > 26 mmol/L] or mixt [pH > 7.45, PaCo_2_ < 35 mmHg, HCO3 > 26 mmol/L])[26, 27].

In addition, to better identify predominant metabolic alkalosis in patients with complex acid–base disorders, we used the Stewart approach, which posits that the body’s pH is primarily determined by the difference in charge between strong ions and weak acids. According to this method, the presence of metabolic alkalosis is indicated by an effective Strong Ion Difference (SIDe) > 40 mEq/L, and/or serum hypoalbuminemia (< 35 g/L). We defined metabolic alkalosis as the predominant acid–base disturbances in the presence of pH > 7.45, SID > 40 mE/L and/or hypoalbuminemia [28, 29].

### Outcomes

Primary outcomes were the relationships among acid–base categories and in-hospital mortality, GOS at discharge [24] and return to baseline premorbid neurologic function at discharge.

Secondary outcome was the distribution of acid–base disturbances according to the Henderson–Hasselbalch equation in the SE patients.

### Statistics

Univariable comparisons were performed by the *χ*2 test for categorical variables. For continuous variables, the Shapiro–Wilk test was used to distinguish between normally and not normally distributed variables.

We assessed relationship among acid–base disturbances according to the Henderson–Hasselbalch equation and primary outcomes first through univariate analyses and then through a binomial regression model considering unbalanced univariate results and variables potentially related to outcomes (age, CCI, SE semeiology, SE potentially fatal etiology, SE duration, STESS, and reliance of invasive therapies).

Then we examined the association between predominant metabolic alkalosis according to the Stewart approach and primary outcomes using both univariate analyses and a binomial regression model.

We assessed laboratory variables associated with the presence of metabolic alkalosis according to according to the Henderson–Hasselbalch equation and the Stewart approach through univariate analysis.

Finally, given the known correlation between sepsis and acid–base unbalance, we performed a subgroup analysis considering only patients with sepsis and evaluating acid–base distribution in this population together with correlation with outcome.

All analyses were performed utilizing the Jamovi software (2.3.21.0 Version).

## Results

### Baseline population features

From 540 identified patients treated for SE during the study period, 44 patients suffered SE after cardiac arrest and were excluded from analysis. Of the remaining 496 patients, BGA during the first 24 h of SE was available in 365 patients, included in the study. Of them, 34 (9%) died during hospital stay, 113 (31%) were discharged with a GOS of 1 to 3, and 160 (44%) did not return to baseline premorbid neurologic function.

Baseline and SE features associated with primary outcomes at univariate analysis are presented in Table 1. Overall, 209 (57%) of SE patients were male, more than 50% required ICU admission, and mean age at SE onset was 61 years. A history of epilepsy was present in 163 (45%). Age and CCI were both associated with in-hospital mortality (*P* = 0.005 and *P* < 0.001, respectively) and no return to premorbid neurologic function at discharge (*P* < 0.001). Mean SE duration and mean STESS values were associated with in-hospital mortality (both *P* < 0.001), GOS 1–3 (*P* < 0.001 and *P* = 0.005, respectively), and no return to premorbid neurologic function at discharge (both *P* < 0.001).

**Table 1:** Univariate analysis assessing relationship between baseline patients and SE features with primary outcomes

	Survival (*N* = 331)	Death (*N* = 34)	*P*	GOS 4–5 (*N* = 252)	GOS 1–3 (*N* = 113)	*P*	RtoB (*N* = 205)	NO RtoB (*N* = 160)	*P*
Patients’ features									
Male sex (%)	189 (57%)	20 (59%)	0.85	141 (56%)	68 (60%)	0.5	98 (61%)	111 (54%)	0.2
Age (M ± SD)	60.1 (18.8)	69.4 (15.4)	**0.005**	60.2 (18.7)	62.8 (18.5)	0.22	57.1 (19.9)	66.0 (15.6)	** < 0.001**
CCI (M ± SD)	3.6 (2.7)	5.6 (3.2)	** < 0.001**	3.6 (2.8)	4.3 (3.0)	**0.026**	3.2 (2.6)	4.6 (3.0)	** < 0.001**
History of epilepsy (%)	156 (47%)	7 (21%)	**0.003**	116 (46%)	47 (42%)	0.4	108 (53%)	55 (34%)	** < 0.001**
SE features (%)									
Duration (M ± SD)	1.1 (1.9)	3.3 (3.7)	** < 0.001**	0.9 (1.6)	2.2 (3.1)	** < 0.001**	0.7 (1.1)	2.0 (3.0)	** < 0.001**
STESS (M ± SD)	2.7 (1.5)	3.9 (1.4)	** < 0.001**	2.7 (1.4)	3.1 (1.6)	**0.009**	2.5 (1.4)	3.2 (1.6)	** < 0.001**
ICU admission (%)	194 (59%)	15 (44%)	0.1	147 (58%)	62 (55%)	0.5	111 (54%)	98 (61%)	0.2
Intubation (%)	190 (58%)	16 (47%)	0.2	144 (57%)	62 (55%)	0.6	112 (55%)	94 (59%)	0.5
SE semeiology									
Motor symptoms (%)	231 (70%)	17 (50%)	**0.019**	169 (67%)	79 (70%)	0.6	143 (70%)	105 (66%)	0.4
Convulsive (%)	175 (53%)	10 (29%)	**0.009**	135 (54%)	50 (42%)	0.1	114 (56%)	71 (44%)	**0.033**
Myoclonic (%)	56 (17%)	7 (21%)	0.6	34 (14%)	29 (26%)	**0.004**	29 (14%)	34 (21%)	0.08
NCSE (%)	100 (30%)	17 (50%)	**0.019**	83 (33%)	34 (30%)	0.6	62 (30%)	55 (34%)	0.4
With coma (%)	24 (7%)	6 (18%)	**0.036**	19 (8%)	11 (10%)	0.5	14 (7%)	16 (10%)	0.3
Without coma (%)	76 (23%)	11 (32%)	0.2	64 (25%)	23 (20%)	0.3	48 (23%)	39 (24%)	0.8
SE etiology									
Acute S (%)	114 (34%)	13 (38%)	0.7	89 (35%)	38 (33%)	0.7	64 (31%)	63 (39%)	0.1
Remote S (%)	130 (39%)	8 (24%)	0.07	91 (36%)	47 (42%)	0.3	82 (40%)	56 (30%)	0.3
Progressive S (%)	45 (14%)	8 (24%)	0.12	36 (14%)	17 (15%)	0.8	24 (12%)	29 (18%)	0.08
Unknown (%)	42 (13%)	5 (15%)	0.7	36 (14%)	11 (10%)	0.2	35 (17%)	8 (12%)	0.007
Potentially fatal (%)	50 (15%)	15 (44%)	** < 0.001**	41 (16%)	24 (21%)	0.2	23 (11%)	42 (26%)	** < 0.001**
Non-mutually exclusive SE etiologies									
Sepsis	34	9	**0.005**	22	21	**0.007**	19	24	0.09
Brain tumors	41	8	0.07	36	13	0.5	23	26	0.6
Old stroke	45	2	0.2	30	17	0.4	23	24	0.3
Acute intracranial hemorrhage	44	10	**0.012**	32	22	0.09	21	33	**0.006**
Acute ischemic stroke	6	3	**0.012**	3	6	**0.019**	2	7	**0.038**
Traumatic brain injury	13	3	0.2	7	9	0.3	7	9	0.3
Alcohol withdrawal	54	0	**0.01**	43	11	0.07	38	16	0.02
Neurodegenerative	10	1	0.9	6	5	0.3	6	5	0.9
Unknown	22	5	0.09	19	8	0.9	15	12	0.9

Bold fond indicates statistical significance

*GOS* Glasgow Outcome Scale, *RtoB* return to baseline, *M* mean *SD* standard deviation, *CCI* Charlson Comorbidity Index, *SE* status epilepticus, *NCSE* nonconvulsive status epilepticus, *STESS* Status Epilepticus Severity Score

SE with prominent motor symptoms was diagnosed in 248 (68%) patients, while 117 (32%) showed NCSE, which was associated with a significantly higher in-hospital mortality rate (*P* = 0.019).

### Acid–base categories according to the Henderson–Hasselbalch equation: univariate analysis

**Fig.1:**
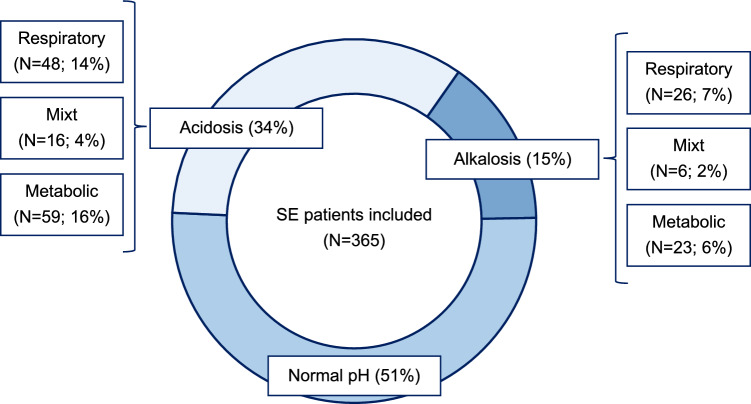
Acid–base category distribution following the Henderson–Hasselbalch approach. *SE* status epilepticus

Acid–base category distribution among SE patients is graphically represented in Fig. 1. Normal mean pH values in the first 24 h of SE were found in 187 (51%) patients. Of them, 6% died during hospital stay, 28% was discharged with a GOS of 1 to 3, and 39% did not regain premorbid neurologic condition at discharge.

Of the 123 (34%) SE patients with a pH < 7.35, respiratory acidosis was found in 48 (14%) patients (in-hospital mortality: 13%; GOS 1–3 at discharge: 25%; no return to baseline neurologic function at discharge: 38%), metabolic acidosis in 59 (16%) patients (in-hospital mortality: 10%; GOS 1–3 at discharge: 28%; no return to baseline neurologic function at discharge: 42%), and mixt acidosis in 16 (4%) patients (in-hospital mortality: 13%; GOS 1–3 at discharge: 25%; no-return to baseline neurologic function at discharge: 44%).

Of the 55 (15%) SE patients with a pH > 7.45, respiratory alkalosis was found in 26 (7%) patients (in-hospital mortality: 10%; GOS 1–3 at discharge: 50%; no-return to baseline neurologic function at discharge: 61%), metabolic alkalosis in 23 (6%) patients (in-hospital mortality: 22%; GOS 1–3 at discharge: 52%; no-return to baseline neurologic function at discharge: 69%), and mixt alkalosis in 6 (2%) (in-hospital mortality: 0%; GOS 1–3 at discharge: 33%; no return to baseline neurologic function at discharge: 67%).

At univariate analysis, a normal pH value was associated to reduced in-hospital mortality (*P* = 0.020), while respiratory alkalosis was associated with GOS 1–3 at discharge (*P* = 0.030). Notably, the presence of metabolic alkalosis was associated with increased in-hospital mortality (*P* = 0.034), GOS 1–3 at discharge (*P* = 0.023), and reduced chance to return to baseline premorbid neurologic function at discharge (*P* = 0.010). Table 2 shows univariate analysis assessing the relationship between acid–base categories and primary outcomes.

**Table 2:** Univariate analysis assessing the relationship between acid–base categories following the Henderson–Hasselbalch approach (with relative mean pH values) and primary outcomes

	pH (M + /SD)	Survival	Death	*P*	GOS 4–5	GOS 1–3	*P*	RtoB	NO RtoB	*P*
Resp Acidosis (*N* = 48)	7.27 (0.08)	42 (87%)	6 (13%)	0.4	41 (75%)	14 (25%)	0.3	34 (62%)	21 (38%)	0.4
Mixt Acidosis (*N* = 16)	7.20 (0.13)	14 (87%)	2 (13%)	0.7	12 (75%)	4 (25%)	0.6	9 (56%)	7 (44%)	0.9
Met acidosis (*N* = 59)	7.28 (0.08)	53 (90%)	6 (10%)	0.8	43 (73%)	16 (27%)	0.5	34 (58%)	25 (42%)	0.8
Normal pH (*N* = 187)	7.39 (0.02)	**176 (94%)**	**11 (6%)**	**0.020**	133 (71%)	54 (29%)	0.4	113 (60%)	74 (40%)	0.1
Resp alkalosis (*N* = 26)	7.47 (0.01)	22 (85%)	4 (15%)	0.3	**13 (50%)**	**13 (50%)**	**0.030**	10 (38%)	16 (62%)	0.1
Mixt alkalosis (*N* = 6)	7.50 (0.022)	6 (100%)	0 (0%)	0.4	4 (67%)	2 (33%)	0.9	2 (33%)	4 (67%)	0.3
Met alkalosis (*N* = 23)	7.48 (0.005)	**18 (78%)**	**5 (22%)**	**0.034**	**11 (48%)**	**12 (52%)**	**0.023**	**7 (30%)**	**16 (70%)**	**0.010**

Bold fond indicates statistical significance

*GOS* Glasgow Outcome Scale, *RtoB* return to baseline, *M* mean, *SD* standard deviation

### Acid–base categories according to the Henderson–Hasselbalch equation: multivariate analysis

**Fig.2:**
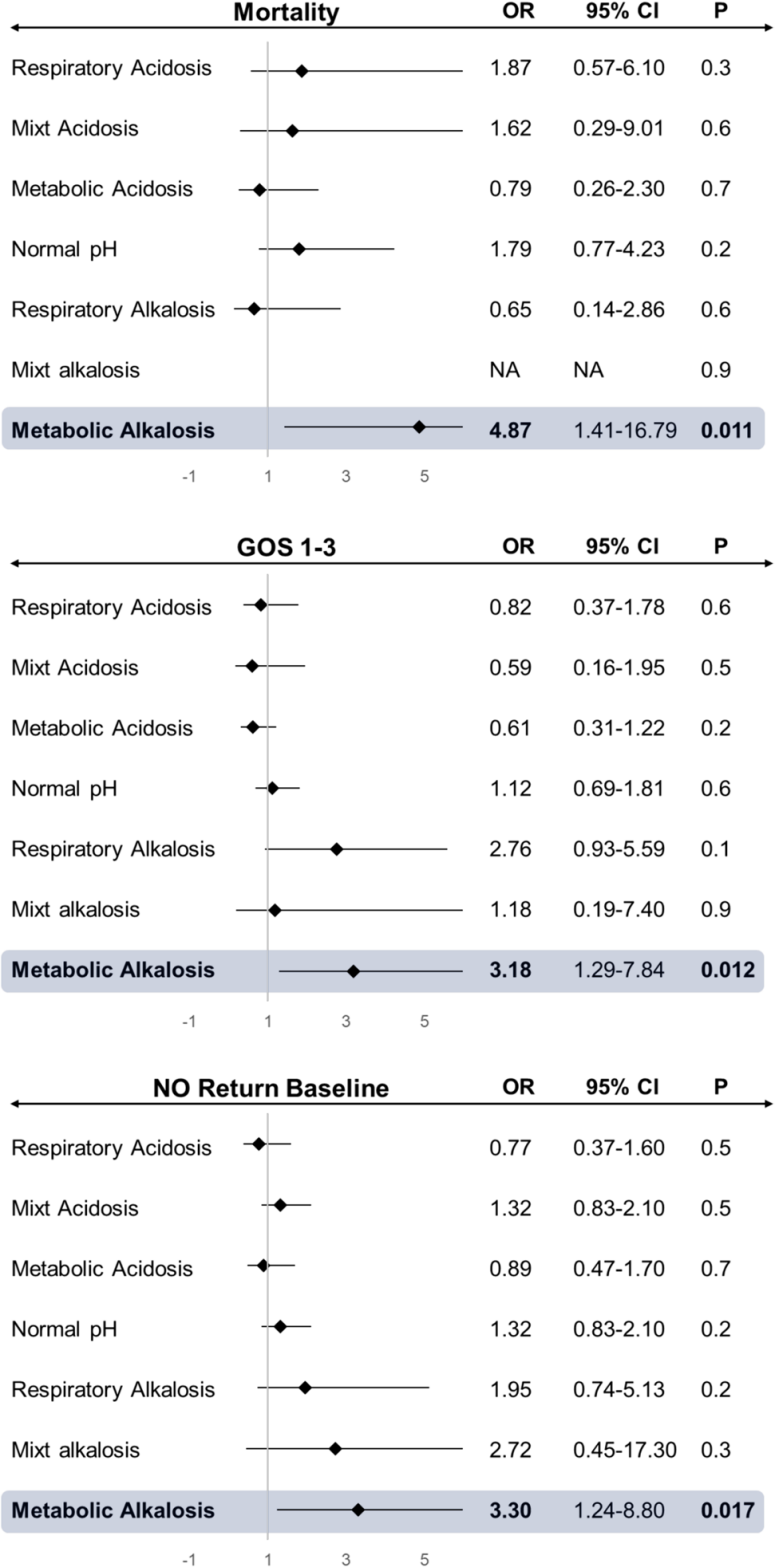
Binomial regression analysis assessing relationship among acid–base categories following the Henderson–Hasselbalch approach and primary outcomes, after adjusting for confounding factors (age, CCI, SE semeiology, SE potentially fatal etiology, SE duration, STESS, and reliance of invasives therapies). *OR* odds ratio, *CI* confidence interval, *GOS* Glasgow Outcome Scale. Bold fond indicates statistical significance

Binomial regression assessing acid–base categories together with age, CCI, SE semeiology, SE potentially fatal etiology, SE duration, STESS, and reliance of invasive therapies revealed that metabolic alkalosis was independently associated with increased risk of in-hospital mortality (*P* = 0.011; odds ratio [OR]:4.87, 95% confidence interval [CI] 1.29–7.84), GOS 1–3 at discharge (*P* = 0.012; OR: 3.18, 95% CI 1.29–7.84), and reduced chance to return to baseline neurologic function (*P* = 0.017; OR: 3.30, CI95% 1.24–8.80). Conversely, all other categories were not related to primary outcomes after correction for other prognostic-related variables. Multivariate analysis results are highlighted in Fig. 2.

### Predominant metabolic alkalosis according to the Stewart equation: univariate and multivariate analyses

Data were available for 315 patients. Of them, 116 (37%) presented a SIDe > 40 mEq/L and/or hypalbuminemia. Predominant metabolic alkalosis as previously defined was present in 30 (9%) patients and was associated to in-hospital mortality (*P* = 0.040), GOS 1–3 at discharge (*P* = 0.001), and an augmented likelihood of not returning to premorbid neurologic function (*P* = 0.005) at univariate analysis. After correction for potential confounders, predominant metabolic alkalosis was still related to worse GOS at discharge (*P* = 0.005; OR: 3.37, 95% CI 1.45–7.82), and reduced chance of returning to baseline (*P* = 0.012; OR: 3.29, CI95% 1.30–8.32).

### Exploratory analyses: laboratory values associated with metabolic alkalosis

Patients with metabolic alkalosis following the Henderson–Hasselbalch equation exhibited significantly lower serum albumin levels (34.0 [± 5.8] g/L vs 39 [± 7.0] g/L; *P* = 0.001) and lower serum potassium levels (3.4 [± 0.4] mmol/L vs 3.9 [± 0.6] mmol/L; *P* < 0.001) compared to other patients, while no significant differences were observed in serum creatinine, serum bilirubin, serum urea, or serum sodium levels.

Patients with predominant metabolic alkalosis following the Stewart equation exhibited significantly lower serum albumin levels (32.0 [± 4.7] g/L vs 40 [± 6.1] g/L; *P* < 0.001), as a direct consequence of its definition, but also lower serum potassium levels (3.5 [± 0.5] mmol/L vs 3.9 [± 0.6] mmol/L; *P* = 0.007) compared to other patients, while no significant differences were observed in serum creatinine, serum bilirubin, serum urea, or serum sodium levels.

### Subgroup analysis: septic patients

Mean pH of the 43 (12%) patients with sepsis was 7.37 [± 0.10]. Eleven (26%) patients showed acidosis (four respiratory, six metabolic, one mixt acidosis), six (14%) patients showed alkalosis (five respiratory, one metabolic), and twenty-six (60%) showed a pH within the normal range. Considering the Stewart approach, five patients (12%) presented a prevalent metabolic alkalosis, significantly related to in-hospital death (*P* = 0.040).

## Discussion

Our study reveals that approximately 50% of SE patients present with an acid–base disorder within the first 24 h of SE according to the Henderson–Hasselbalch approach.

About 35% of patients experienced respiratory and/or metabolic acidosis, likely directly related to respiratory disfunction and/or lactate production [11, 13, 14]. The antiepileptic effect of acidosis is well established: neuronal excitability and seizure activity are strongly suppressed by a decrease in brain and blood pH [7, 8], with some anticonvulsants, such as acetazolamide, acting reducing extracellular brain pH [30]. However, the prognostic role of acidosis in critically ill patients remains debated. Some studies associate acidosis to higher mortality [31, 32], while others find no relationship with outcome [33], or even a correlation to better prognosis [34, 35]. In our study, we did not find a clear association between acidosis and outcomes, but a larger sample size may be needed to detect small differences.

In the first 24 h of SE, 15% of patients showed alkalosis, split evenly between metabolic and respiratory causes. The pathogenesis of alkalosis in SE is less clear, and likely influenced by the overall pathophysiological context. Alkalosis, especially metabolic alkalosis, has been recognized as a common acid–base disturbance in critically ill patients [36, 37], increasing with invasive therapies like mechanical ventilation, gastric aspiration, and intravenous infusions [38]. In our cohort, we assessed pH only in the very first stage of SE and only a small percentage of patients experienced alkalosis, with no relationship to intensive care unit (ICU) admission or invasive therapies, suggesting their non-primary role in this context.

An increase in pH is associated with the generation of spontaneous interictal spikes in animal models, potentially contributing to the interictal–ictal transition [39]. In this context, hyperventilation-induced alkalosis has long been used to induce seizure activity/electroencephalogram abnormalities as an activation test [40].

Several reports highlighted that the presence of metabolic alkalosis is associated with longer ICU stays and increased mortality in patients with various diseases [41–43]. In our cohort, metabolic alkalosis at admission was a strong indicator of higher mortality, worse GOS, and a lower likelihood of returning to baseline even after correction for potential confounders.

Our findings were further validated using the Stewart approach, which, despite being more complex and less user-friendly for clinical practice, offers a more detailed and precise assessment of acid–base disorders, particularly in critical care settings [28, 29]. According to the Stewart method, predominant metabolic alkalosis was associated with all primary outcomes in univariate analysis and remained significantly linked to a reduced likelihood of good functional outcomes after binomial regression.

Metabolic alkalosis may directly contribute to poor outcomes or be an indicator of other adverse factors. Some studies suggest that metabolic alkalosis directly impacts outcomes by reducing respiratory input, inhibiting hemoglobin dissociation and causing electrolytes imbalance [41, 44]. We analyzed the number of patients with metabolic alkalosis according to the Henderson–Hasselbalch approach who normalized their pH levels within the subsequent 24 h: of 17 patients with available data, the normalization of pH had no clear impact on prognosis, but the extremely small sample size warrants caution in interpretation.

While alkalosis may contribute directly (e.g., through respiratory depression), other factors likely play a significant role in reducing the likelihood of good outcome for alkalosis patients in our cohort. We found a significant correlation between metabolic alkalosis, hypoalbuminemia, and lower serum potassium. The association between alkalosis and hypoalbuminemia is well established [45], as well as the bidirectional relationship among metabolic alkalosis and hypokalemia [46].

Hypoalbuminemia is a well-known predictor of poor outcomes across various conditions, including cardiovascular disease, cancer, and sepsis [47–50], and it might be considered as a reliable biomarker of overall patient’s resources to survive SE. Similarly, low serum potassium has been associated with adverse outcomes in critically ill patients, including an increased risk of respiratory insufficiency, with a reduced capacity to wean from mechanical ventilation [51].

Our study has several limitations. First, as a retrospective cohort study covering nearly a decade, it may have been affected by changes in the management of SE patients over time. In addition, we did not account for pre-hospital management, which could have influenced pH levels in the early stages of SE or at hospital admission. Furthermore, we were unable to evaluate long-term outcomes due to a lack of data. Another limitation is the variability in the timing of pH assessments relative to SE onset and hospital admission, which our data do not capture. However, BGA are routinely performed during the initial assessment after hospital admission, and for patients with multiple ABG assessments, only the first one was considered.

Finally, while it is known that respiratory-induced alkalosis might facilitate seizure occurrence and respiratory-induced acidosis might elevate the seizure threshold promoting seizure cessation, our study did not directly focus on SE duration in relation to pH [8–10]. However, no pH categories were related to SE duration in our study, and the association between metabolic alkalosis and outcomes remained significant after adjusting for several confounders, including SE duration. This makes it very unlikely that the observed association between metabolic alkalosis and outcomes is based on SE duration.

## Conclusion

In our cohort, approximately half of the patients exhibited acid–base disturbances within the first 24 h of SE, according to the Henderson–Hasselbalch approach. Although less common, metabolic alkalosis was strongly associated with in-hospital mortality and severe impairment at discharge, findings that were further confirmed using the Stewart approach.

It is unlikely that alkalosis originates from SE itself, and it remains unclear whether correcting pH values can improve outcomes. Alkalosis likely results from factors such as low serum albumin and electrolyte imbalances, which are themselves associated with poor prognosis. Robust prospective studies are needed to determine whether prompt recognition and aggressive treatment of alkalosis, hypoalbuminemia, and electrolyte imbalances can improve outcomes in SE patients.

## Data Availability

The data that support the findings of this study are available from the corresponding author upon reasonable request.
